# Randomised controlled trial comparing efficacy and safety of high versus low Low-Molecular Weight Heparin dosages in hospitalized patients with severe COVID-19 pneumonia and coagulopathy not requiring invasive mechanical ventilation (COVID-19 HD): a structured summary of a study protocol

**DOI:** 10.1186/s13063-020-04475-z

**Published:** 2020-06-26

**Authors:** Marco Marietta, Paola Vandelli, Pasquale Mighali, Roberto Vicini, Valeria Coluccio, Roberto D’Amico, on behalf of COVID-19 HD Study Group, Daniela Aschieri, Lucio Brugioni, Enrico Clini, Mauro Codeluppi, Davide Imberti, Andrea Magnacavallo, Marianna Meschiari, Cristina Mussini, Sergio Orlando, Giovanni Pinelli, Antonello Pietrangelo, Luca Sarti, Matteo Silva

**Affiliations:** 1 Dipartimento Oncologia ed Ematologia, Azienda Ospedaliero-Universitaria di Modena, Ospedale Policlinico, Modena, Italy; 2 Servizio Formazione, Ricerca e Innovazione, Azienda Ospedaliero-Universitaria di Modena, Ospedale Policlinico, Modena, Italy; 3 Cattedra di Statistica Medica, Dipartimento di Scienze Mediche e Chirurgiche Materno-Infantili e dell’Adulto, Università di Modena e Reggio Emilia, Modena, Italy

**Keywords:** COVID-19, Randomised controlled trial, Protocol, Low-molecular weight heparin, Enoxaparin, Pneumonia, Coagulopathy

## Abstract

**Objectives:**

To assess whether high doses of Low Molecular Weight Heparin (LMWH) (i.e. Enoxaparin 70 IU/kg twice daily) compared to standard prophylactic dose (i.e., Enoxaparin 4000 IU once day), in hospitalized patients with COVID19 not requiring Invasive Mechanical Ventilation [IMV], are:

- more effective in preventing clinical worsening, defined as the occurrence of at least one of the following events, whichever comes first:
  1. Death
  2. Acute Myocardial Infarction [AMI]
  3. Objectively confirmed, symptomatic arterial or venous thromboembolism [TE]
  4. Need of either:
    - Continuous Positive Airway Pressure (Cpap) or Non-Invasive Ventilation (NIV) or
    - IMV in patients who at randomisation were receiving standard oxygen therapy
  5. IMV in patients who at randomisation were receiving non-invasive mechanical ventilation
- Similar in terms of major bleeding risk

**Trial design:**

Multicentre, randomised controlled, superiority, open label, parallel group, two arms (1:1 ratio), in-hospital study.

**Participants:**

Inpatients will be recruited from 7 Italian Academic and non-Academic Internal Medicine Units, 2 Infectious Disease Units and 1 Respiratory Disease Unit.

**Inclusion Criteria (all required):**

1. Age > 18 and < 80 years
2. Positive SARS-CoV-2 diagnostic (on pharyngeal swab of deep airways material)
3. Severe pneumonia defined by the presence of at least one of the following criteria:
  - Respiratory Rate ≥25 breaths /min
  - Arterial oxygen saturation≤93% at rest on ambient air
  - PaO2/FiO2 ≤300 mmHg
4. Coagulopathy, defined by the presence of at least one of the following criteria:
  - D-dimer >4 times the upper level of normal reference range
  - Sepsis-Induced Coagulopathy (SIC) score >4
5. No need of IMV

**Exclusion Criteria:**

1. Age <18 and >80 years
2. IMV
3. Thrombocytopenia (platelet count < 80.000 mm3)
4. Coagulopathy: INR >1.5, aPTT ratio > 1.4
5. Impaired renal function (eGFR calculated by CKD-EPI Creatinine equation < 30 ml/min)
6. Known hypersensitivity to enoxaparin
7. History of heparin induced thrombocytopenia
8. Presence of an active bleeding or a pathology susceptible of bleeding in presence of anticoagulation (e.g. recent haemorrhagic stroke, peptic ulcer, malignant cancer at high risk of haemorrhage, recent neurosurgery or ophthalmic surgery, vascular aneurysms, arteriovenous malformations)
9. Concomitant anticoagulant treatment for other indications (e.g. atrial fibrillation, venous thromboembolism, prosthetic heart valves)
10. Concomitant double antiplatelet therapy
11. Administration of therapeutic doses of LMWH, fondaparinux, or unfractionated heparin (UFH) for more than 72 hours before randomization; prophylactic doses are allowed
12. Pregnancy or breastfeeding or positive pregnancy test
13. Presence of other severe diseases impairing life expectancy (e.g. patients are not expected to survive 28 days given their pre-existing medical condition)
14. Lack or withdrawal of informed consent

**Intervention and comparator:**

**Control Group (Low-Dose LMWH):** patients in this group will be administered Enoxaparin (Inhixa®) at standard prophylactic dose (i.e., 4000 UI subcutaneously once day).

**Intervention Group (High-Dose LMWH):** patients in this group will be administered Enoxaparin (Inhixa®) at dose of 70 IU/kg every 12 hours, as reported in the following table. This dose is commonly used in Italy when a bridging strategy is required for the management of surgery or invasive procedures in patients taking anti-vitamin K oral anticoagulants

**Table:** 

Body Weight (kg)	Enoxaparin dose every 12 hours (IU)
<50	2000
50-69	4000
70-89	6000
90-110	8000
>110	10000

The treatment with Enoxaparin will be initiated soon after randomization (maximum allowed starting time 12h after randomization). The treatment will be administered every 12 hours in the intervention group and every 24 hours in the control group. Treatments will be administered in the two arms until hospital discharge or the primary outcomes detailed below occur.

**Main outcomes:**

**Primary Efficacy Endpoint:**

Clinical worsening, defined as the occurrence of at least one of the following events, whichever comes first:

1. Death
2. Acute Myocardial Infarction [AMI]
3. Objectively confirmed, symptomatic arterial or venous thromboembolism [TE]
4. Need of either:
  - Continuous Positive Airway Pressure (Cpap) or Non-Invasive Ventilation (NIV) or
  - IMV in patients who at randomisation were in standard oxygen therapy by delivery interfaces
5. Need for IMV, in patients who at randomisation were in Cpap or NIV

Time to the occurrence of each of these events will be recorded.

Clinical worsening will be analysed as a binary outcome as well as a time-to-event one.

**Secondary Efficacy Endpoints:**

Any of the following events occurring within the hospital stay

1. Death
2. Acute Myocardial Infarction [AMI]
3. Objectively confirmed, symptomatic arterial or venous thromboembolism [TE]
4. Need of either:
  - Continuous Positive Airway Pressure (Cpap) or Non-Invasive Ventilation (NIV) or
  - IMV in patients who at randomisation were in standard oxygen therapy by delivery interfaces
5. Need for IMV in patients who at randomisation were in Cpap or NIV
6. Improvement of laboratory parameters of disease severity, including:
  o D-dimer level
  o Plasma fibrinogen levels
  o Mean Platelet Volume
  o Lymphocyte/Neutrophil ratio
  o IL-6 plasma levels

**Mortality at 30 days:**

Information about patients’ status will be sought in those who are discharged before 30 days on Day 30 from randomisation.

Time to the occurrence of each of these events will be recorded.

Each of these events will be analysed as a binary outcome and as a time-to-event one.

**Primary safety endpoint:**

Major bleeding, defined as an acute clinically overt bleeding associated with one or more of the following:

- Decrease in haemoglobin of 2 g/dl or more;
- Transfusion of 2 or more units of packed red blood cells;
- Bleeding that occurs in at least one of the following critical sites [intracranial, intraspinal, intraocular (within the corpus of the eye; thus, a conjunctival bleed is not an intraocular bleed), pericardial, intra-articular, intramuscular with compartment syndrome, or retroperitoneal];
- Bleeding that is fatal (defined as a bleeding event that was the primary cause of death or contributed directly to death);
- Bleeding that necessitates surgical intervention

Time to the occurrence of each of these events will be recorded.

Each of these events will be analysed as a binary outcome and as a time-to-event one.

**Secondary safety endpoint:**

Clinically Relevant non-major bleeding, defined as an acute clinically overt bleeding that does not meet the criteria for major and consists of:

1. Any bleeding compromising hemodynamic
2. Spontaneous hematoma larger than 25 cm2, or 100 cm2 if there was a traumatic cause
3. Intramuscular hematoma documented by ultrasonography
4. Epistaxis or gingival bleeding requiring tamponade or other medical intervention
5. Bleeding from venipuncture for >5 minutes
6. Haematuria that was macroscopic and was spontaneous or lasted for more than 24 hours after invasive procedures
7. Haemoptysis, hematemesis or spontaneous rectal bleeding requiring endoscopy or other medical intervention
8. Any other bleeding requiring temporary cessation of a study drug.

Time to the occurrence of each of these events will be recorded.

Each of these events will be analysed as a binary outcome and as a time-to-event one.

**Randomisation:**

Randomisation (with a 1:1 randomisation ratio) will be centrally performed by using a secure, web-based system, which will be developed by the Methodological and Statistical Unit at the Azienda Ospedaliero-Universitaria of Modena. Randomisation stratified by 4 factors: 1) Gender (M/F); 2) Age (<75/≥75 years); 3) BMI (<30/≥30); 4) Comorbidities (0-1/>2) with random variable block sizes will be generated by STATA software. The web-based system will guarantee the allocation concealment.

Blinding (masking)

The study is conceived as open-label: patients and all health-care personnel involved in the study will be aware of the assigned group.

**Numbers to be randomised (sample size):**

The target sample size is based on the hypothesis that LMWH administered at high doses versus low doses will significantly reduce the risk of clinical worsening. The overall sample size in this study is expected to be 300 with 150 in the Low-Dose LMWH control group and 150 in the High-Dose LMWH intervention group, recruited over 10-11 months. Assuming an alpha of 5% (two tailed) and a percentage of patients who experience clinical worsening in the control group being between 25% and 30%, the study will have 80% power to detect at least 50% relative reduction in the risk of death between low and high doses of heparin.

**Trial Status:**

Protocol version 1.2 of 11/05/2020.

Recruitment start (expected): 08/06/2020

Recruitment finish (expected): 30/04/2021

Trial registration

EudraCT 2020-001972-13, registered on April 17th, 2020

Full protocol

The full protocol is attached as an additional file, accessible from the Trials website (Additional file 1). In the interest in expediting dissemination of this material, the familiar formatting has been eliminated; this Letter serves as a summary of the key elements of the full protocol.

## Supplementary information

**Additional file 1.** Full protocol.

## Data Availability

The study data will be collected during the entire study period in a dedicated electronic Case Report Form (eCRF) provided by the Steering Committee (SC). Data will be collected and stored on the hospital server, which will be protected by password to prevent unintentional modification or deletion.

