# Supplementary material for: Randomised controlled trial comparing efficacy and safety of high versus low Low-Molecular Weight Heparin dosages in hospitalized patients with severe COVID-19 pneumonia and coagulopathy not requiring invasive mechanical ventilation (COVID-19 HD): a structured summary of a study protocol
Source: Trials. 2020 Jun 26;21:574. doi: 10.1186/s13063-020-04475-z (PMC7316577; doi:10.1186/s13063-020-04475-z)
Supplement: Supplementary file 1 — Additional file 1. Full protocol. [file 13063_2020_4475_MOESM1_ESM.docx]

CLINICAL STUDY PROTOCOL

| Study Title: | **Randomised controlled trial comparing efficacy and safety of high versus low Low-Molecular Weight Heparin dosages in hospitalised patients with severe COVID-19 pneumonia and coagulopathy not requiring invasive mechanical ventilation (COVID-19 HD)** |
| --- | --- |
| Short title: | **COVID-19 HD** |
| EudraCT N°: | **2020-001972-13** |
| Sponsor: | Azienda Ospedaliero-Universitaria di Modena |
| Study Coordinator: | Marco Marietta, MD  Dipartimento Oncologia ed Ematologia  Azienda Ospedaliero-Universitaria di Modena  Via del Pozzo 71 – 41124 Modena – Italy  Phone: +393487331047  e-mail: marco.marietta@unimore.it |
| **Financial Support:**  **Contact Information:** | Azienda Ospedaliero-Universitaria di Modena  Via del Pozzo 71 – 41124 Modena – Italy  Marco Marietta, MD  Dipartimento Oncologia ed Ematologia  Azienda Ospedaliero-Universitaria di Modena  Via del Pozzo 71 – 41124 Modena – Italy  Phone: +393487331047  e-mail: marco.marietta@unimore.it |
| Investigational Product | enoxaparin (Inhixa®) |
| Clinical Study Phase: | III |
| Version: | 1.2 |
| Issue Date: | 11^th^ – May- 2020 |
|  |  |

**STUDY SYNOPSIS**

| **Protocol Title** | Randomised controlled trial comparing efficacy and safety of high versus low Low-Molecular Weight Heparin dosages in hospitalised patients with severe COVID-19 pneumonia and coagulopathy not requiring invasive mechanical ventilation (COVID-19 HD) |  |
| --- | --- | --- |
| **Clinical Trial Phase** | III |  |
| **Study Site** | Azienda Ospedaliero-Universitaria di Modena  Ospedale Policlinico di Modena  Via del Pozzo 71 – 41124 Modena – Italy |  |
| **Background and Rationale** | In December 2019, a cluster of acute respiratory illnesses caused by SARS-CoV-2 virus occurred in Wuhan, Hubei Province, China. The disease has rapidly spread from Wuhan to many other countries worldwide, soon becoming a global health emergency.  Pneumonia is the most frequent and serious complication of COVID-19, the disease that results from SARS-CoV-2 infection.  The pathogenesis of SARS-CoV-2 infection in humans remains unclear, although it is very likely that the most severe manifestations of COVID-19 may be linked to host-pathogen interaction immune mechanisms.  Both coagulation factors and platelets are directly implicated in the modulation of the host immune response, displaying proinflammatory functions independent of their hemostatic effects.  Heparin, being an anticoagulant that also possesses immunomodulatory properties, represents a promising drug for lowering immune response and hypercoagulability observed in COVID-19 patients. Its use at prophylactic doses has been shown to be associated with a reduced 28-day mortality in severe COVID-19 patients with coagulopathy. However, no evidence is still available as to whether higher doses of heparin can improve the prognosis of more severe COVID-19 patients without affecting the safety aspects related to bleeding. |  |
| **Study Period** | June 2020 – June 2021 |  |
| **Study population** | Hospitalized patients with severe COViD-19 pneumonia and coagulopathy not requiring invasive mechanical ventilation. |  |
| **Primary Study Objective(s)** | To assess whether high doses of Low Molecular Weight Heparin (LMWH) (ie. Enoxaparin 70 IU/kg twice daily) compared to standard prophylactic dose (ie, Enoxaparin 4000 IU once day) are:  a) more effective to prevent clinical worsening, defined as the occurrence of at least one of the following events, whichever comes first, during hospital stay:   1. Death 2. Acute Myocardial Infarction [AMI] 3. Objectively confirmed, symptomatic arterial or venous thromboembolism [TE] 4. Need for either non-invasive - Continuous Positive Airway Pressure (Cpap) or Non-Invasive Ventilation (NIV) - or invasive mechanical ventilation for patients who are in standard oxygen therapy by delivery interfaces at randomisation 5. Need for invasive mechanical ventilation for patients who are in non-invasive mechanical ventilation at randomisation 6. Similar in terms of major bleeding risk during hospital stay |  |
| **Secondary Study**  **Objectives** | To assess whether high doses of Low Molecular Weight Heparin (LMWH) (ie. Enoxaparin 70 IU/kg twice daily) compared to standard prophylactic dose (ie, Enoxaparin 4000 IU once day) differ in terms of the following events occurring during hospital stay:   1. Laboratory parameters of disease severity, including: 2. D-dimer level 3. Plasma fibrinogen levels 4. Mean Platelet Volume 5. Lymphocyte/Neutrophil ratio 6. IL-6 plasma levels 7. Clinically relevant non-major bleeding 8. Death during hospital stay and at 30 days 9. Acute Myocardial Infarction [AMI] 10. Objectively confirmed, symptomatic arterial or venous thromboembolism [TE] 11. Need for either non-invasive - Continuous Positive Airway Pressure (Cpap) or Non-Invasive Ventilation (NIV) - or invasive mechanical ventilation for patients, who are in standard oxygen therapy by delivery interfaces at randomisation 12. Need for invasive mechanical ventilation for patients, who are in non-invasive mechanical ventilation at randomisation | |
| **Statistical Considerations**  **& Sample Size** | **Statistical Considerations**  The efficacy will be assessed by estimating the difference between the two treatment arms in the proportion of participants experiencing clinical worsening during their hospital stay.  Safety will be assessed by estimating the difference between the two treatment arms in the proportion of participants who experience at least one bleeding event (major or non-major) during their hospital stay.  Relative risk (RR) will be used as measure of association, which will be reported with its 95% confidence interval.  The differences between the two arms in the variation of:   1. D-dimer level 2. Plasma fibrinogen levels   3) Mean platelet Volume  4) Lymphocyte/Neutrophil ratio  5) IL-6 plasma levels  at 3 time points (at randomization, at 48 hours and 96 hours) will be assessed.  These outcomes will be compared by using their mean values and summary indicators.  Overall survival, survival free from any cardiovascular event, such as Acute Myocardial Infarction [AMI], Objectively confirmed, symptomatic arterial or venous thromboembolism [TE] and survival free from need for either non-invasive or invasive mechanical ventilation will be also estimated. They will be reported by using Kaplan-Maier curves and compared by using hazard ratio (HR).  **Sample size**  300 participants will be needed to detect a relative reduction of 50% of clinical worsening. We hypothesized that the percentage of participants who experience clinical worsening in the LMWH low dose group would be around 25-30% and set an alpha error of 0.05 and beta-error of 0.20. | |
| **Treatment Schedule** | **Control group (Low Dose):** Enoxaparin (Inhixa®) 4000 IU subcutaneously once daily.  **Intervention group (High dose**): Enoxaparin (Inhixa®) 70 IU/kg subcutaneously twice daily.  Participants will receive the dose of Enoxaparin according to the protocol until the occurrence of at least one of the following events:   1. One of the events included in the primary outcome 2. Hospital discharge 3. Any adverse events and clinical condition requiring interruption of the scheduled intervention according to the judgement of the physician in charge | |
| **Inclusion criteria** | Patients admitted to the hospital with COVID-19 confirmed by PCR test on throat swab samples, and with severe pneumonia plus coagulopathy, defined as the presence of at least one clinical and one laboratory criteria (see table below)   \| **Clinical criteria**  **(at least one)** \| **Laboratory criteria and SIC score**  **(at least one)** \| \| --- \| --- \| \| 1) Respiratory Rate ≥ 25 breaths /min \| 1) D-dimer > 4 times the upper level of normal reference range \| \| 2) Arterial oxygen saturation ≤ 93% at rest in room air \| 2) Sepsis-Induced Coagulopathy (SIC) score > 4 \| \| 3) PaO2/FiO2 ≤ 300 mmHg \|  \| | |

| **Main Exclusion Criteria** | 1. Age < 18 and > 80 years 2. Invasive ventilation 3. Thrombocytopenia (platelet count < 80.000 mm^3^) 4. Coagulopathy: INR >1.5, aPTT ratio >1.4 5. Impaired renal function (eGFR by CKD-EPI Creatinine equation < 30 ml/min) 6. Known hypersensitivity to enoxaparin 7. History of heparin induced thrombocytopenia 8. Presence of an active bleeding or a pathology susceptible of bleeding in presence of anticoagulation (e.g. recent haemorrhagic stroke, peptic ulcer, malignant tumors at high risk of haemorrhages, recent neurosurgery or ophthalmic surgery, vascular aneurysms, arteriovenous malformations) 9. Concomitant anticoagulant treatment for other indications (e.g. atrial fibrillation, venous thromboembolism, prosthetic heart valves) 10. Concomitant double antiplatelet therapy 11. Administration of therapeutic doses of LMWH, fondaparinux, or unfractionated heparin (UFH) for more than 72 hours before randomization; prophylactic doses are allowed 12. Pregnancy or breastfeeding or positive pregnancy test 13. Presence of other severe diseases impairing life expectancy (e.g. patients are not expected to survive 28 days given their pre-existing medical condition) 14. Lack or withdrawal of informed consent |
| --- | --- |

**INDEX**

**Protocol Signature Form 2**

**Study synopsis 3**

**List of abbreviations 9**

**Roles and responsibilities 10**

**1.0 Introduction 12**

1.1 Background and rationale 12

1.1.1 Benefits on the use of Low Molecular Weight Heparin 12

1.2 Study objective 13

1.3 Trial design 13

**2.0 Methods: participants, Interventions and outcomes 14**

2.1 Study setting 14

2.2 Eligibility criteria 14

2.2.1 Inclusion Criteria 14

2.2.2 Exclusion criteria 14

2.3 Interventions 15

2.3.1 Duration of treatments 15

2.3.2 Concomitant medications 16

2.3.3 Criteria for discontinuing or modifying allocated interventions 16

2.3.4 Strategies to improve adherence to protocols 16

2.4 Outcomes 16

2.4.1 Primary efficacy endpoint 16

2.4.2 Secondary efficacy endpoints 17

2.4.3 Primary safety endpoint 17

2.4.4 Secondary safety endpoint 17

2.4.5 Risk/benefit ratio 18

2.4.6 Physiological and Processes of care outcomes 18

2.5 Participant timeline 18

2.6 Sample size 18

2.7 Recruiment 19

**3.0 Methods: assignment of interventions 19**

3.1 Allocation and blinding 19

**4.0 Methods: data collection, management and analysis 19**

4.1 Data collection and management 20

4.2 Statistical Methods 20

4.3 Data monitoring 20

4.4 Analyses ad interim 20

**5.0 Safety criteria evaluation 21**

5.1 Safety profile 21

5.2 Adverse events (AE) and serious adverse events (SAE) 21

5.2.1 Definition of an AE 21

5.2.2 Definition of a SAE 21

5.3 Recording of AEs and SAEs 22

5.3.1 Evaluating AEs and SAEs 22

5.3.1.1 Assessment of intensity 23

5.3.1.2 Assessment of causality 23

5.3.1.3. Follow-up of AEs and SAEs 23

5.4 Regulatory reporting requirements for adverse events 23

**6.0 Ethics and dissemination 24**

6.1 Ethics review 24

6.2 Ethical conduction of the study 24

6.3 Written informed consent 25

6.4 Subject data protection 25

6.5 Audits and inspections 25

6.6 Declaration of interests 25

6.7 Dissemination policy 25

**7.0 Bibliography** 27

APPENDIX 1: List of centres 29

APPENDIX 2: Flow-chart of the study 31

APPENDIX 3: Sequential Organ Failure Assessment (SOFA) score 33

APPENDIX 4: Study timeline 34

**LIST OF ABBREVIATIONS**

| AE:  ADR:  AIFA:  aPTT  BMI  CC  Cpap  COVID-19:  DSMB:  EC:  eCRF:  GCP:  H:  Hb:  HCT:  IL-6  IU  HR:  INR:  LMWH:  MRC:  NIV  PI:  PLT:  PT:  RR: | Adverse event  Adverse drug reaction  Italian medicines agency  Adjusted Partial Pro-thrombin time  Body Mass Index  Coordination Centre  Continuous Positive Airway Pressure  Corona Virus 19 Disease  Data Safety Monitoring Board  Ethical Committee  electronic Case Report Form  Good Clinical Practice  hours  Haemoglobin  Hematocrit  Interleukin-6  International Units  Hearth rate  International normalised ratio  Low Molecular Weight Heparin  Medical Research Council  Non-Invasive Ventilation  Principal Investigator  Platelets  Pro-thrombin time  Respiratory rate | SARS-Cov2  SC:  SIC  SOFA  UFH:  VTE:  WHO: | New Corona Virus  Steering Committee  Sepsis Induced Coagulopathy  Sequential Organ Failure Assessment  Unfractionated Heparin  Venous Thrombo Embolism  World Health Organization |
| --- | --- | --- | --- |

| Roles and responsibilities: | | | |
| --- | --- | --- | --- |
| **Principal investigator and coordinating centre** | Marco Marietta, MD | Dipartimento Oncologia ed Ematologia  Azienda Ospedaliero-Universitaria di Modena  Ospedale Policlinico  Via del Pozzo 71 – 41124 Modena – Italy  Phone: +393487331047  e-mail: marco.marietta@unimore.it |  |
| **Statistical design, data management and analysis** | Roberto D’Amico, PHD | Unità di Supporto Statistico Metodologico per la Ricerca Clinica  Azienda Ospedaliero-Universitaria di Modena.  Università di Modena and Reggio Emilia.  Via del Pozzo 71 – 41124 Modena – Italy  Phone: +393333664682  e-mail: roberto.damico@unimore.it |  |
| **Steering Committee** | Enrico Clini, MD, PHD | Respiratory Diseases Unit, Department of Medical and Surgical Sciences, University Hospital of Modena and Center for Rare Lung Diseases  University of Modena Reggio Emilia, Modena, Italy  Ospedale Policlinico  Via del Pozzo 71 – 41124 Modena – Italy  e-mail: enrico.clini@unimore.it |  |
|  | Mauro Codeluppi | Infectious Disease Unit  Onco-Hematology Department  Hospital of Piacenza  Via Taverna 49 - 29121 Piacenza Italy  e-mail: m.codeluppi@ausl.pc.it |  |
|  | Davide Imberti | Internal Medicine Unit  Medical Department  Hospital of Piacenza  Via Taverna 49 - 29121 Piacenza Italy  e-mail: d.imberti@ausl.pc.it |  |
|  | Cristina Mussini | Infectious Diseases Clinics  Azienda Ospedaliero Universitaria Policlinico and University of Modena and Reggio Emilia  Ospedale Policlinico  Via del Pozzo 71 – 41124 Modena – Italy  e-mail: cristina.mussini@unimore.it |  |
|  | Antonello Pietrangelo | Dipartimento di Medicina Interna Generale, d’Urgenza e Post-Acuzie  Azienda Ospedaliero-Universitaria di Modena  Via del Pozzo 71 – 41124 Modena – Italy  e-mail: [antonello.pietrangelo@unimore.it](mailto:antonello.pietrangelo@unimore.it) |  |
|  | Luca Sarti | Medicina Interna d'Urgenza e Area Critica  Dipartimento di Medicina Interna Generale, d’Urgenza e Post-Acuzie  Ospedale Civile di Baggiovara  via Pietro Giardini, 1355 – 41126 Modena  e-mail:sarti.luca@aou.mo.it |  |
| **Co-investigator** | Valeria Coluccio, MD | Dipartimento Oncologia ed Ematologia  Azienda Ospedaliero-Universitaria di Modena  Via del Pozzo 71 – 41124 Modena – Italy  Phone: +390594225031  e-mail:  coluccio.valeria@policlinico.mo.it |  |
|  | Marianna Meschiari, MD | Infectious Diseases Clinics  Azienda Ospedaliero Universitaria Policlinico and University of Modena and Reggio Emilia  Ospedale Policlinico  Via del Pozzo 71 – 41124 Modena – Italy  e-mail: mariannameschiari1209@gmail.com |  |

This is an investigator-initiated study. The Steering Committee will take responsibility for study design and data analysis and will operate actions necessary to guarantee that the trial is conducted in accordance with procedures described in this document and good clinical practice. The funders will have no role in study design, data collection, management, analysis, data interpretation, manuscript writing, or in the decision to submit manuscripts for publication.

**1.0 INTRODUCTION**

**1.1 BACKGROUND AND RATIONALE**

In December 2019, a cluster of acute respiratory illnesses caused by SARS-CoV-2 virus occurred in Wuhan, Hubei Province, China. The disease has rapidly spread from Wuhan to many other countries worldwide, soon becoming a global health emergency.

Although most patients have mild manifestations and good prognosis after infection, some patients develop severe symptoms and die from multiple organ complications. Pneumonia is the most frequent and serious complication of COVID-19, the disease that results from SARS-CoV-2 infection [1,2].

The pathogenesis of SARS-CoV-2 infection in humans remains unclear, although it is very likely that the most severe manifestations of COVID-19 may be linked to host-pathogen interaction immune mechanisms. [3]

SARS-CoV-2 infection seems to induce in most critical cases an excessive and aberrant hyper-inflammatory host immune response that is associated with a so-called "cytokine storm", characterized by plasma increase of infection-related biomarkers and of many cytokines and chemokines documented in many observational studies. [4]. A pro-thrombotic derangement of the haemostatic system has been reported in most severe forms of COVID-19 infections [5-10].

This finding is consistent with the activation of a coagulative cascade primed by inflammatory stimuli already observed in many other forms of sepsis. [11] Indeed, several studies have demonstrated the close interconnection between thrombosis and inflammation, two processes mutually reinforcing each other [12-14]. Both coagulation factors [15] and platelets [16] are directly implicated in the modulation of the host immune response, displaying proinflammatory functions independent of their haemostatic effects.

From a clinical perspective, the extent of the derangement in coagulation parameters in patients affected by severe COVID-19 pneumonia was associated to a poor prognosis. [17,18] In these patients, Low Molecular Weight Heparin (LMWH) at doses registered for prevention of venous thromboembolism (VTE), i.e. Enoxaparin 4000 IU subcutaneously daily, has been proven to be associated with a reduced risk of death [19] and is currently recommended by World Health Organization [20] and by several Scientific Societies [18-25].

However, some issues about the use of LMWH in COVID-19 pts require further clarification.

First, the reported incidence of venous thromboembolism in COVID-19 patients is strikingly variable, ranging from 0% to about 8% in general wards [26,27], and from 16% to 35% in the ICU setting, often despite adequate LMWH prophylaxis [28-35].

This finding prompted some Authors to raise the question whether the pulmonary vessels occlusions observed in COVID-19 patients result from embolization of a deep-venous thrombosis or from a thrombotic microangiopathy affecting the pulmonary vascular bed [28].

On this regard, it has been suggested that the diffuse bilateral pulmonary inflammation observed in COVID-19 is associated with a novel pulmonary-specific pathophysiological entity termed pulmonary intravascular coagulopathy (PIC), distinct from Disseminated Intravascular Coagulation. [36]

LMWH has also been shown to protect glycocalyx from shedding, displaying anti-inflammatory and immunomodulatory properties. [37] Of note, in vitro and in vivo experimental studies have shown that human coronaviruses utilize heparin sulphate proteoglycans for attachment to target cells [38], suggesting a role for heparin in the therapeutic armamentarium against COVID-19 [39].

On these grounds, it is conceivable that doses of LMWH higher than those used for VTE prevention in acutely ill medical patients, might improve anti-inflammatory activity, mitigate cytokines storm and improve the disease prognosis.

However, evidence is not yet available as to whether higher doses of heparin can improve the prognosis of more severe COVID-19 patients without affecting the safety related to bleeding.

Indeed, the paper from Tang and colleagues showed that the use of LMWH reduces the 28-day mortality only in patients with the most severe degree of coagulopathy, as assessed by a Sepsis-Induced Coagulopathy (SIC) score >4 or d-dimer levels >6-fold the upper limit of normal, whereas a non-significant trend towards a negative effect of the treatment was observed in the less severely affected ones [19].

This finding highlights the need for a very careful selection of patients’ population in designing trials on this setting, because of the narrow margin on the risk-benefit ratio.

We report here the design of the COVID-19 HD study, a randomized trial comparing high doses of Low Molecular Weight Heparin (LMWH) (ie. Enoxaparin 70 IU/kg twice daily) with standard prophylactic dose (ie, Enoxaparin 4000 IU once day) for the treatment of severe pneumonia and coagulopathy in hospitalised patients with COVID19 not requiring invasive mechanical ventilation (IMV).

**1.1.3 Benefits of the use LMWH**

As reported in the previous section, the use of LMWH or Unfractionated Heparin (UFH) at prophylactic doses has been shown to be associated with a reduced mortality at 28 days in COVID-19 patients with severe pneumonia and Sepsis Induced Coagulopathy (SIC) score ≥4 (40.0% vs 64.2%, P=0.029), or D-dimer > 6-fold of upper limit of normal (32.8% vs 52.4%, P=0.017 [11]. This finding is consistent with the well-known immune-modulatory properties and protective action of glycocalyx from shedding displayed by heparin. [15].

However, no evidence is available about the clinical question of whether higher doses of LMWH improve the prognosis of more severe COVID-19 patients.

**1.2 STUDY OBJECTIVE**

To assess whether high doses of Low Molecular Weight Heparin (LMWH) (i.e. Enoxaparin 70 IU/kg twice daily) compared to standard prophylactic dose (i.e., Enoxaparin 4000 IU once day) are:

1. more effective to prevent clinical worsening, defined as the occurrence of at least one of the following events, whichever comes first:
2. Death
3. Acute Myocardial Infarction [AMI]
4. Objectively confirmed, symptomatic arterial or venous thromboembolism [TE]
5. Need for either non-invasive - Continuous Positive Airway Pressure (Cpap) or Non-Invasive Ventilation (NIV) - or invasive mechanical ventilation for patients, who are in standard oxygen therapy by delivery interfaces at randomisation
6. Need for invasive mechanical ventilation for patients, who are in non-invasive mechanical ventilation at randomisation
7. Similar in terms of major bleeding risk

**1.3 TRIAL DESIGN**

This is a multicentre, randomised controlled, open label, investigator sponsored, two arms study.

Patients who satisfy all inclusion criteria and no exclusion criteria and have signed written informed consent, will be randomly assigned to one of the two treatment groups in a 1:1 ratio.

**2.0** **METHODS: PARTICIPANTS, INTERVENTIONS AND OUTCOMES**

**2.1 STUDY SETTING**

The study will involve 7 Italian Academic and non-Academic Internal Medicine Units, 2 Infectious Disease Units, 1 Respiratory Disease Unit.

See list of centres in APPENDIX 1.

**2.2 ELIGIBILITY CRITERIA**

**2.2.1 Inclusion Criteria (all required)**

1. Age > 18 and < 80 years
2. Positive SARS-CoV-2 diagnostic (on pharyngeal swab of deep airways material)
3. Severe pneumonia defined by the presence of at least one of the following criteria:
   1. Respiratory Rate ≥25 breaths /min
   2. Arterial oxygen saturation≤93% at rest on ambient air
   3. PaO2/FiO2 ≤300 mmHg
4. Coagulopathy, defined by the presence of at least one of the following criteria:
   1. D-dimer >4 times the upper level of normal reference range
   2. Sepsis-Induced Coagulopathy (SIC) score >4
5. No need for invasive mechanical ventilation
   - 1. **Exclusion Criteria**
6. Age <18 and >80 years
7. Invasive mechanical ventilation

3. Thrombocytopenia (platelet count < 80.000 mm^3^)

4. Coagulopathy: INR >1.5, aPTT ratio > 1.4

5. Impaired renal function (eGFR calculated by CKD-EPI Creatinine equation < 30 ml/min)

6. Known hypersensitivity to enoxaparin

7. History of heparin induced thrombocytopenia

8. Presence of an active bleeding or a pathology susceptible of bleeding in presence of anticoagulation (e.g. recent haemorrhagic stroke, peptic ulcer, malignant cancer at high risk of haemorrhage, recent neurosurgery or ophthalmic surgery, vascular aneurysms, arteriovenous malformations)

9. Concomitant anticoagulant treatment for other indications (e.g. atrial fibrillation, venous thromboembolism, prosthetic heart valves).

10. Concomitant double antiplatelet therapy

11. Administration of therapeutic doses of LMWH, fondaparinux, or unfractionated heparin (UFH) for more than 72 hours before randomization; prophylactic doses are allowed

12. Pregnancy or breastfeeding or positive pregnancy test

13. Presence of other severe diseases impairing life expectancy (e.g. patients are not expected to survive 28 days given their pre-existing medical condition)

14. Lack or withdrawal of informed consent

**2.3 INTERVENTIONS**

Patients who satisfy all inclusion criteria and do not have any exclusion criteria and have signed written informed consent, will be randomly assigned to a Low-Dose LMWH group (Control Group) or High-Dose LMWH group (Intervention Group).

The study is conceived as open-label: patients and all health-care personnel involved in the study will be aware of the assigned group. The treatments will be initiated as soon as possible after randomization (maximum allowed starting time 12h after randomization).

**Control Group** (Low-Dose LMWH): patients in this group will be administered Enoxaparin (Inhixa®) at standard prophylactic dose (i.e., 4000 UI subcutaneously once day).

**Intervention Group** (High-Dose LMWH): patients in this group will be administered Enoxaparin (Inhixa®) at dose of 70 IU/kg every 12 hours, as reported in the following table.

| **Body Weight (kg)** | **Enoxaparin dose every 12 hours (IU)** |
| --- | --- |
| <50 | 2000 |
| 50-69 | 4000 |
| 70-89 | 6000 |
| 90-110 | 8000 |
| >110 | 10000 |

The treatment with Enoxaparin will be initiated as soon as possible after randomization (maximum allowed starting time 12h after randomization). The treatment will be administered every 12 hours in the intervention group and every 24 hours in the control group.

**Duration of treatments**

Patients allocated to the two arms will maintain the doses of Enoxaparin, as stated in the protocol, until:

1. hospital discharge

or

1. when at least one of the following events occurs:
2. Acute Myocardial Infarction [AMI]
3. Objectively confirmed, symptomatic arterial or venous thromboembolism [TE]
4. Need for either non-invasive - Continuous Positive Airway Pressure (Cpap) or Non-Invasive Ventilation (NIV) - or invasive mechanical ventilation for patients, who are in standard oxygen therapy by delivery interfaces at randomisation
5. Need for invasive mechanical ventilation for patients, who are in non-invasive mechanical ventilation at randomisation
6. Major bleeding
7. Any adverse events and clinical condition requiring interruption of the scheduled intervention according to the judgement of the physician in charge
8. Death

The decision about what type and dose of antithrombotic treatment to administer, after the interruption of assigned dose of Enoxaparin, will be left to clinical judgement of the physicians in charge.

Any information about the type and dose of antithrombotic treatments administered after the interruption of the assigned dose of Enoxaparin will be collected until the hospital discharge or death.

**Duration of follow-up**

Each patient will be followed-up until hospital discharge. Information about the status (dead/alive) of patients who are discharged from hospital before 30 days will be sought on Day 30 from randomisation.

**Concomitant medications**

Concomitant diseases or conditions will be treated according to the principles of Good Clinical Practice and according to clinical judgement of the attending physician. There will be no restrictions on concomitant treatments for the underlying disease to be given to patients enrolled in this study.

Any relevant health intervention received by participants 24 hours before the randomisation and during the study period will be recorded.

**Criteria for discontinuing or modifying allocated interventions**

LMWH administration will start at the moment of randomization and will last until the hospital discharge, or up to the occurrence of any event included in the primary outcome.

Participants who during their hospital stay develop any new clinical condition, which require anticoagulant or antiplatelet treatment, such as atrial fibrillation and/or substitution of heart valves, will discontinue the allocated intervention and will be excluded from the study.

Discontinuation or modification of allocated interventions can also be warranted by the occurrence of one of the following events: any AE, including clinically relevant, non-major bleeding or clinically significant laboratory abnormality that, in the opinion of the Investigator, warrants the subject’s discontinuation, temporary or permanent, of study protocol-directed care.

Reasons for discontinuation or modification of allocated interventions and their dates will be collected and reported in the eCRF.

**Strategies to improve adherence to protocols**

The site PIs will take primary responsibility for training local staff and for ensuring that protocol compliance is achieved.

**2.4 OUTCOMES**

**2.4.1 Primary Efficacy Endpoint:**

Clinical worsening, defined as the occurrence of at least one of the following events, whichever comes first:

1. Death
2. Acute Myocardial Infarction [AMI]
3. Objectively confirmed, symptomatic arterial or venous thromboembolism [TE]
4. Need for either non-invasive - Continuous Positive Airway Pressure (Cpap) or Non-Invasive Ventilation (NIV) - or invasive mechanical ventilation for patients, who are in standard oxygen therapy by delivery interfaces at randomisation
5. Need for invasive mechanical ventilation for patients, who are in non-invasive mechanical ventilation at randomisation

Time to the occurrence of each of these events will be recorded.

Clinical worsening will be analysed as a binary outcome as well as a time-to-event one.

**2.4.2 Secondary Efficacy Endpoints:**

**Any of the following events occurring within the hospital stay**

1. Death
2. Acute Myocardial Infarction [AMI]
3. Objectively confirmed, symptomatic arterial or venous thromboembolism [TE]
4. Need for either non-invasive - Continuous Positive Airway Pressure (Cpap) or Non-Invasive Ventilation (NIV) - or invasive mechanical ventilation for patients, who are in standard oxygen therapy by delivery interfaces at randomisation
5. Need for invasive mechanical ventilation for patients, who are in non-invasive mechanical ventilation at randomisation
6. Improvement of laboratory parameters of disease severity, including:
   - D-dimer level
   - Plasma fibrinogen levels
   - Mean Platelet Volume
   - Lymphocyte/Neutrophil ratio
   - IL-6 plasma levels

**Mortality at 30 days**

Information about patients’ status will be sought in those who are discharged before 30 days on Day 30 from randomisation.

Time to the occurrence of each of these events will be recorded.

Each of these events will be analysed as a binary outcome and as a time-to-event one.

**2.4.3 Primary safety endpoint:**

Major bleeding, defined as an acute clinically overt bleeding associated with one or more of the following:

- Decrease in haemoglobin of 2 g/dl or more;
- Transfusion of 2 or more units of packed red blood cells;
- Bleeding that occurs in at least one of the following critical sites [intracranial, intraspinal, intraocular (within the corpus of the eye; thus, a conjunctival bleed is not an intraocular bleed), pericardial, intra-articular, intramuscular with compartment syndrome, or retroperitoneal];
- Bleeding that is fatal (defined as a bleeding event that was the primary cause of death or contributed directly to death);
- Bleeding that necessitates surgical intervention

Time to the occurrence of each of these events will be recorded.

Each of these events will be analysed as a binary outcome and as a time-to-event one.

- - 1. **Secondary safety endpoint:**

Clinically Relevant non-major bleeding, defined as an acute clinically overt bleeding that does not meet the criteria for major and consists of:

1. Any bleeding compromising hemodynamics
2. Spontaneous hematoma larger than 25 cm^2^, or 100 cm^2^ if there was a traumatic cause
3. Intramuscular hematoma documented by ultrasonography
4. Epistaxis or gingival bleeding requiring tamponade or other medical intervention
5. Bleeding from venipuncture for >5 minutes
6. Hematuria that was macroscopic and was spontaneous or lasted for more than 24 hours after invasive procedures
7. Hemoptysis, hematemesis or spontaneous rectal bleeding requiring endoscopy or other medical intervention
8. Any other bleeding requiring temporary cessation of a study drug.

Time to the occurrence of each of these events will be recorded.

Each of these events will be analysed as a binary outcome and as a time-to-event one.

**2.4.4 Risk/benefit Ratio**

Recently published data have shown that the use of low-dose heparin in patients admitted to hospital with COVID-19 and severe pneumonia and coagulopathy, reduces mortality from about 60% to about 30%.

We assume that higher doses of LMWH in these patients might be more effective than low doses in reducing the risk of clinical worsening. At the same time, we are aware that the administration of high doses of LMWH might be associated with a higher risk of bleeding. However, we think that the possible increased risk of major bleeding could be negligible, as Wein et al [16] reported a risk of major bleeding between 1.4% and 2.29% with low doses of LMWH, whereas Pengo et al [17] reported a 2.7% risk when LMWH was given at high doses.

We think that on these premises, the risk/benefit of the intervention is favourable for severe COVID-19 patients and allows us to consider the study worth carrying out.

**2.4.3 Physiological and Processes of care outcomes**

- Vital signs such as systolic and diastolic arterial pressure, heart rate (HR), respiratory rate (RR) and systemic body temperature will be recorded daily from inclusion until hospital discharge (censored day 28);
- Routine laboratory test parameters for organ function assessment: blood cells count, coagulative parameters (INR, PT, aPTT, fibrinogen, D-dimer), parameters for liver (AST, ALT, bilirubin) and renal function (creatinine) and interleukin 6 (IL-6) will be recorded at least at the moment of randomization, after 48 and 96 hours, then according to clinical judgement of the attending physician until hospital discharge.

**2.5 PARTICIPANT TIMELINE**

See APPENDIX 2 for a timeline-scheme of the trial.

**2.6 SAMPLE SIZE**

The target sample size is based on the hypothesis that LMWH administered at high doses versus low doses will significantly reduce the risk of clinical worsening. The overall sample size in this study is expected to be 300 with a 1:1 randomisation ratio. Assuming an alpha of 5% (two tailed) and a percentage of patients who experience clinical worsening in the control group being between 25% and 30%, the study will have 80% power to detect at least 50% relative reduction in the risk of death between low and high doses of heparin.

**2.7 RECRUITMENT**

All centres participating in this study are experienced in investigator-initiated studies. On the basis of a conservative estimate, each of the 10 participating sites is expected to see around 4 eligible patients per month (40 patients/month). Assuming that 80% of those patients will be enrolled, the recruitment of 300 participants will be completed in around 10-11 months. This period could be shorter if other centres join the study.
Every month, recruitment status will be evaluated, and a newsletter will be disseminated, including any practical, clinical or scientific issues that have arisen.

**3.0 METHODS: ASSIGNEMENT OF INTERVENTIONS**

**3.1 Allocation and blinding**

Randomisation will be centrally performed by using a secure, web-based system, which will be developed by the Methodological and Statistical Unit at the Azienda Ospedaliero-Universitaria of Modena. Randomisation stratified by 4 factors: 1) Gender (M/F); 2) Age (<75/≥75 years); 3) BMI (<30/≥30); 4) Co-morbidities (0-1/>2) with random variable block sizes will be generated by STATA software. The web-based system will guarantee the allocation concealment. The physicians involved in the study, once they have obtained the patient’s consent, will connect to the website and will register the patient that meets inclusion criteria. The system will also allow the randomisation of the patient and the arm to which he/she is allocated will appear on the monitor. The physician who performed the randomisation will communicate which dose the patient will receive to the colleagues who are in charge of administering LMWH. The information collected during the patient follow-up will be entered on the website and stored on the Hospital’s server. The local coordinator will be in charge of training the personnel involved in the study according to what is stated in the protocol. The study is conceived as open label, i.e. the patients and all healthcare personnel will be aware of the group allocation.

**4.0 METHODS: DATA COLLECTION, MANAGEMENT, ANALYSIS AND MONITORING**

**4.1 Data collection and management**

Every patient who meets the inclusion criteria will be included and randomised in the two groups. The study data will be collected during the entire study period in a dedicated electronic Case Report Form (eCRF).

The eCRF will be provided by the Steering Committee (SC) with proper options to minimize data entry errors: the datasheet will incorporate un-amendable fixed intervals of values (for continuous variables) and pre-defined coding system (for binary or categorical variables). Data entry will be performed and double-checked by a dedicated team of researchers. Data will be collected and stored on the hospital server, which will be protected by password to prevent unintentional modification or deletion. Data related to the study will be stored for eventual further analysis or study purpose for 10 years after the end of the study.

All the data about the included patients will be recorded in an eCRF by trained researchers.

Demographic information, symptoms and co-morbidities, will be registered at the inclusion. During the study duration, the following clinical and laboratory parameters will be evaluated and recorded according to the timeline schedule reported in APPENDIX 2:

- Vital signs such as systolic and diastolic arterial pressure, heart rate (HR), respiratory rate (RR) and systemic body temperature will be recorded daily from inclusion until hospital discharge;

- Routine laboratory test parameters for organ function assessment: blood cells count, coagulative parameters (INR, PT, aPTT, fibrinogen, D-dimer), parameters for liver (AST, ALT, bilirubin) and renal function (creatinine) and interleukin 6 (IL-6) will be recorded at least at the moment of randomisation, after 48 and 96 hours, then according to clinical judgement of the attending physician until hospital discharge.

**4.2 Statistical Methods**

All patients enrolled in the study will be entered in the full analysis set independently of his/her treatment time. The intention to treat population will be considered for primary analysis. A descriptive statistical analysis will be carried out to summarise every relevant variable. At baseline, categorical data will be summarised by using counts and percentages, whilst continuous variables will be presented using the number of patients, mean, standard deviation, median, minimum, and maximum. Comparison between binary outcomes will be performed by using Relative Risks (RRs), whereas for continuous data, the difference of means (DMs) will be used. Comparisons involving time to event data will be displayed by using Kaplan-Maier survival curves and summarised by Hazard Ratios (HRs).

All measures of association will be presented with their 95% confidence interval. A result will be considered as statistically significant if its p-values will be less than 0.05 (5%).

The analyses will be performed by using STATA software.

**4.3 Data Monitoring**

An independent Data Safety Monitoring Board (DSMB), consisting of experts in clinical research, in haematology and in statistics/methodology, will be established before patient enrolment. The DSMB Charter will be prepared by the SC and signed by the members of the DSMB before the trial commences. The DSMB will have access to the summary of all results and will consider the appropriateness of the sample size, of the efficiency and of the quality of the data collection system, and eventual occurrence of suspected protocol-related adverse events. The DSMB will have the right to stop the trial for safety reasons.

**4.4 Analyses ad interim**

Considering the statistical approach, an interim analysis is planned after the randomisation of 150 patients (50% of sample size) for the double objective of monitoring safety and verifying the accuracy of the assumptions made for sample size estimation, regarding the event rate of the primary outcome in relation to the anticipated survival benefit. With the interim analysis we will be able to evaluate whether the data suggests that there is substantial superiority of one treatment. The obtained results will be evaluated by the DSMB and by the SC and, in case of significant differences in survival among the groups, all patients will be switched to the most promising treatment.

1. **SAFETY CRITERIA EVALUATION**
   1. **Safety profile**

Data on safety profile, nature, incidence and severity of adverse events (AEs) and serious adverse events (SAEs) will be collected. Any reason for drug interruption, reduction and discontinuation will be collected. Toxicities will be graded using NCI Common Terminology Criteria for adverse Events (CTCAE) version 4.03.

- 1. **Adverse events (AE) and serious adverse events (SAE)**

The investigator is responsible for the detection and documentation of AE and SAE events, according to the criteria defined in this protocol. During the safety evaluation, the Investigator or site staff will be responsible for detecting, documenting and reporting AEs and SAEs, as detailed in both this section of the protocol and in the AE/SAE section of the CRF.

- - 1. **Definition of an AE**

An AE is defined as any untoward medical occurrence in a patient, temporarily associated with the use of a medicinal product, whether or not it is considered related to the medicinal product. An AE can therefore be any unfavourable and unintended sign (including an abnormal laboratory finding), symptom, or disease (new or exacerbated) temporarily associated with the use of a medicinal product. For marketed medicinal products, this also includes failure to produce expected benefits (i.e. lack of efficacy), abuse or misuse.

Examples of an AE include:

- Significant or unexpected worsening or exacerbation of the condition/indication under study.
- Exacerbation of a chronic or intermittent pre-existing condition including either an increase in frequency and/or intensity of the condition.
- New conditions detected or diagnosed after investigational product administration even though it may have been present prior to the start of the study.
- Signs, symptoms, or the clinical sequelae of a suspected interaction.
- Signs, symptoms, or the clinical sequelae of a suspected overdose of either investigational product or a concurrent medication (overdose per se should not be reported as an AE/SAE).

Examples of an AE do not include:

- Medical or surgical procedure (e.g., endoscopy, appendectomy); the condition that leads to the procedure is an AE.
- Situations where an untoward medical occurrence did not occur (social and/or convenience admission to a hospital).
  - 1. **Definition of an SAE**

An SAE is defined as any untoward medical occurrence that, at any dose:

1. Results in death
2. Is life-threatening

Note: The term 'life-threatening' in the definition of 'serious', refers to an event in which the subject was at risk of death at the time of the event. It does not refer to an event, which hypothetically might have caused death, if it were more severe.

1. Requires hospitalization or prolongation of existing hospitalization

Note: In general, hospitalization signifies that the subject has been detained (usually involving at least an overnight stay) at the hospital or emergency ward for observation and/or treatment that would not have been appropriate in the physician’s office or in an outpatient setting. Complications that occur during hospitalization are AEs. If a complication prolongs hospitalization or fulfils any other serious criteria, the event is serious. When in doubt as to whether “hospitalization” occurred, or was necessary, the AE should be considered serious. Hospitalization for elective treatment of a pre-existing condition that did not worsen from the baseline is not considered an AE.

1. Results in disability/incapacity

Note: The term disability means a substantial disruption of a person’s ability to conduct normal life functions. This definition is not intended to include experiences of relatively minor medical significance such as uncomplicated headache, nausea, vomiting, diarrhoea, influenza, and accidental trauma (e.g. sprained ankle) which may interfere or prevent everyday life functions but do not constitute a substantial disruption.

1. Is a congenital anomaly/birth defect.

Medical or scientific judgement should be exercised in deciding whether reporting is appropriate in other situations, such as important medical events that may not be immediately life-threatening or result in death or hospitalization but may jeopardize the subject or may require medical or surgical intervention to prevent one of the other outcomes listed in the above definition. These should also be considered serious. Examples of such events are invasive or malignant cancers, intensive treatment in an emergency room or at home for allergic bronchospasm, blood dyscrasias or convulsions that do not result in hospitalization, or development of drug dependency or drug abuse.

- 1. **Recording of AEs and SAEs**

When an AE/SAE occurs, it is the responsibility of the investigator to review all documentation (e.g. hospital progress notes, laboratory, and diagnostics reports) relative to the event. The investigator will then record all relevant information regarding an AE or SAE on the eCRF. It is not acceptable for the investigator to send photocopies of the subject’s medical records in lieu of completion of the appropriate AE or SAE CRF pages. The investigator will attempt to establish a diagnosis of the event based on signs, symptoms, and/or other clinical information. In such cases, the diagnosis should be documented as the AE/SAE and not the individual signs/symptoms. Any AEs or SAEs occurring during the study must be documented in the subject's medical records and on the appropriate page of the eCRF. Each AE or SAE is to be recorded individually. All AEs which occur during the course of the study should be recorded in the eCRF. Information on the AE must be recorded on a specific AE form and forwarded to the DSMB. The SC and the DSMB will evaluate the adverse events occurred during the study.

- - 1. **Evaluating AEs and SAEs**
       1. **Assessment of intensity**

The investigator will make an assessment of intensity of each AE and SAE reported. In this protocol, the intensity of AEs and SAEs will be graded on a scale of 1 to 5 according the National Cancer Institute (NCI) Common Toxicity Criteria for Adverse Events (CTCAE) Version 4.03 and are available at [http://evs.nci.nih.gov/ftp1/CTCAE/CTCAE_4.03_2010-06- 14_QuickReference_5x7.pdf](http://evs.nci.nih.gov/ftp1/CTCAE/CTCAE_4.03_2010-06-%2014_QuickReference_5x7.pdf).

For SAEs, the maximum intensity (or grade) will be reported in the eCRF. For non-serious AEs, each change in intensity (or grade) will be reported in the eCRF.

- - - 1. **Assessment of causality**

The investigator is obliged to assess the relationship between the study medical product and the occurrence of each AE/SAE. The investigator will use clinical judgement to determine the relationship. Alternative causes, such as natural history of the underlying diseases, concomitant therapy, other risk factors, and the temporary relationship of the event to the study drugs will be considered and investigated. There may be situations when an SAE has occurred, and the investigator has minimal information to include in the initial report. However, it is very important that the investigator always makes an assessment of causality for every event prior to transmission of the SAE form to the Coordinating Centre. The investigator may change his/her opinion of causality in light of follow-up information, amending the SAE eCRF accordingly. The causality assessment is one of the criteria used when determining regulatory reporting requirements. The investigator will provide the assessment of causality as per instructions on the SAE form in the Investigators File.

- - - 1. **Follow-Up of AEs and SAEs**

After the initial AE/SAE report, the investigator is required to proactively follow each subject and provide further information on the subject’s condition. All AEs and SAEs documented at a previous visit/contact and are designated as ongoing, will be reviewed at subsequent visits/contacts. AEs that are ongoing with a toxicity of Grade 3 or 4, or have a relationship to study drug that is suspected (Reasonable Possibility) will be queried for resolution at study conclusion and at approximately 30 days after the last dose of study. Once resolved, the appropriate AE/SAE CRF page(s) will be updated. The investigator will ensure that follow-up includes any supplemental investigations as may be indicated to elucidate the nature and/or causality of the AE or SAE. This may include additional laboratory tests or investigations, or consultation with other health care professionals. New or updated information will be recorded on the originally completed SAE form in the Investigator’s File, with all changes signed and dated by the Investigator.

- 1. **Regulatory reporting requirements for adverse events**

The Principal Investigator at Coordinating Centre (CC) has a legal responsibility to notify, as appropriate, both the local regulatory authority and other regulatory agencies about the safety of a product under clinical investigation. Prompt notification of SAEs by the Investigator to the appropriate project contact for SAE receipt is essential so that legal obligations and ethical responsibilities towards the safety of other subjects are met. The Principal Investigator at CC, or responsible person according to local requirements, will comply with the applicable local regulatory requirements related to the reporting of SAEs to regulatory authorities and the Independent Ethics Committee (IEC). In particular, all the Suspected Unexpected Serious Adverse Reactions (SUSARs) that occur while on treatment and within 30 days since the last investigational drug administration, and that have a suspected relationship to study’s drug (Reasonable Possibility) will be notified with an urgency procedure to the local regulatory Agency (AIFA) and IEC with the following timelines:

- SUSARs that are considered life-threatening: notification within 7 days.
- SUSARs that are not considered life-threatening: notification within 15 days.

The notification with urgency procedure is not required for SAEs that are expected with the drugs used in the protocol, and for non-serious AEs, both expected and unexpected. For these events (expected SAEs and AEs), the CC will notify the local regulatory agency and IECs by an annual safety report. The CC is responsible for providing all the investigators involved in the trial with appropriate timelines, and all the safety information relevant for patient safety.

1. **ETHICS**

The study will be conducted in line with the protocol, the Declaration of Helsinki (1964) and subsequent amendments and updates (Fortaleza, Brazil, October 2013). Moreover, it is the responsibility of the investigator to ensure that the study will be done in line with the requirements of Good Clinical Practice (GCP) and the applicable regulatory requirements.

**6.1 Ethics review**

The entire study protocol, including informative material for the patients and modules for the informed consent, will be evaluated by the Ethics Committee (EC) of the National Institute for Infectious Diseases “Lazzaro Spallanzani”, Rome, Italy, which is the National Ethics Committee for evaluation of clinical trials on human drugs in COVID-19 patients. The study will not start before obtaining a favourable opinion from the EC, the Competent Authority Authorization and any other authorization required by local regulation. Every intention to modify any element of the original protocol after the first approval will be promptly notified to the EC and will be applied only after its written authorization.

Investigators will be responsible for submitting any amendments to the protocol to the EC.

Any modifications to the protocol which may impact on the conduct of the study, may affect patient safety, including changes of study objectives, study design, patient population, sample size, study procedures or significant administrative aspects will require a formal amendment to the protocol. Such amendments will be agreed and approved by the Ethics Committee of the National Institute for Infectious Diseases “Lazzaro Spallanzani”, Rome, Italy, as the National Ethics Committee for evaluation of clinical trials on human drugs in COVID-19 patients and the health authorities prior to implementation, in accordance with local regulation. Administrative changes of the protocol are minor corrections and/or clarifications that have no effect on the way the study is to be conducted. These administrative changes will be documented in a memorandum.

**6.2 Ethical conduct of the study**

The study was designed and will be implemented and performed in accordance with the ICH Harmonized Tripartite Guidelines for Good Clinical Practice, with applicable local regulations (including European Directive 2001/20/EC), and with the ethical principles laid down in the Declaration of Helsinki.

- 1. **Written informed consent**

The Investigator at each Centre will ensure that the subject is given full and adequate oral and written information about the nature, purpose, possible risks and benefits of the study. Subjects must also be notified that they are free to discontinue from the study at any time. The subject’s signed and dated informed consent must be obtained prior to conduct any procedure specific for the study. The original signed Written Informed Consent Form must be stored, and a copy must be given to the patient.

- 1. **Subject data protection**

The Written Informed Consent Form will explain that the study data will be stored on a centralised hospital server, maintaining confidentiality in accordance with national data legislation. The Investigator must ensure anonymity of the patients; all computer data will be identified by subject initial and number only. Enrolment log must be kept strictly confidential to enable patient identification at the site.

- 1. **Audits and inspections**

The Principal Investigator and the SC will provide all the necessary information and material to the participating centres in order to standardize all the protocol-related procedures and to avoid unexpected variability between centres. A preliminary audit between the CC and the satellite centres will be performed before starting the recruitment phase to offer as much consensus and homogeneity as possible. Printed and electronic informative material (complete original protocol, informed consent modules, informative modules for patients and relatives, recruitment checklist, graphic timeline of interventions and visits, order list for physicians and nurses) will be distributed to every Centre.

Source data/documents must be available to inspections by the designee or Health Authorities.

- 1. **Declaration of interest**

The study participants declare no financial and/or other conflicts of interest related to the study.

- 1. **Dissemination policy**

The Circ. Min. Health N° 6 of 09/02/2002 obliges each researcher who gets any results of interest to public health, to publish the results within 12 months from the end of the study. All the patients will freely agree or disagree to participate in the study in the belief that the results will be useful to improve knowledge about their pathologies, for health benefit from themselves or other patients. To respect their will and in the maximum interest of honest clinical research, the investigators agree on the need to ensure the wide publication and diffusion of their results in a consistent and responsible way under their responsibility. The Study Coordinator is the official data owner. The Study Coordinator has the right to present methods and results of the study at public symposia and conferences. The principal publications from the trial will be in the name of Investigators with full credit assigned to all collaborating investigators and institutions.

**7.0 BIBLIOGRAPHY**

1. Qin, C. *et al.* Dysregulation of immune response in patients with COVID-19 in Wuhan, China. *Clin. Infect. Dis. Off. Publ. Infect. Dis. Soc. Am.* (2020) doi:10.1093/cid/ciaa248.
2. Wu, C. *et al.* Risk Factors Associated With Acute Respiratory Distress Syndrome and Death in Patients With Coronavirus Disease 2019 Pneumonia in Wuhan, China. *JAMA Intern. Med.* (2020) doi:10.1001/jamainternmed.2020.0994.
3. Wang, D. *et al.* Clinical Characteristics of 138 Hospitalized Patients With 2019 Novel Coronavirus–Infected Pneumonia in Wuhan, China. *JAMA* **323**, 1061–1069 (2020).
4. Jackson SP, Darbousset R,Schoenwaelder SM, et al. Thromboinflammation: challenges of therapeutically targeting coagulation and other host defense mechanisms. Blood. 2019; 133:906-918.
5. Iba T, Levy JH. Inflammation and thrombosis: roles of neutrophils, platelets and endothelial cells and their interactions in thrombus formation during sepsis. J Thromb Haemost 2018; 16: 231–41.
6. Claushuis TAM, de Stoppelaar SF, Stroo I, Roelofs JJTH, et al. Thrombin contributes to protective immunity in pneumonia-derived sepsis via fibrin polymerization and platelet–neutrophil interactions. J Thromb Haemost 2017; 15: 744–57.
7. Burzynski LC, Humphry M, Pyrillou K et al. The Coagulation and Immune Systems Are Directly Linked through the Activation of Interleukin-1α by Thrombin. Immunity. 2019; 50:1033-1042.e6.
8. Chen J, Li X, Li L et al. Coagulation factors VII, IX and X are effective antibacterial proteins against drug-resistant Gram-negative bacteria. Cell Research 2019; 29:711–724.
9. Assinger A, Schrottmaier WC, Salzmann M, Rayes J Platelets in Sepsis: An Update on Experimental Models and Clinical Data. Front Immunol. 2019; 10:1687.
10. Tang N, Li D, Wang X, Sun Z. Abnormal coagulation parameters are associated with poor prognosis in patients with novel coronavirus pneumonia.J Thromb Haemost. 2020 Feb 19.
11. Tang N, Bai H, Chen X, Gong J, Li D, Sun Z. Anticoagulant treatment is associated with decreased mortality in severe coronavirus disease 2019 patients with coagulopathy. J Thromb Haemost. 2020 Mar 27. doi: 10.1111/jth.14817.
12. Tcachil J, Tang N, Gando S et al. ISTH interim guidance on recognition and management of coagulopathy in COVID-19. Journal Thomb Haemost first published: 25 March 2020 doi: 10.1111/jth.14810
13. Marietta M, Ageno W, Artoni A et al. COVID-19 and haemostasis: a position paper from Italian Society on Thrombosis and Haemostasis (SISET). Blood Transfus. 2020 Apr 8. doi: 10.2450/2020.0083-20
14. Li X, Ma X. The role of heparin in sepsis: much more than just an anticoagulant. British Journal of Haematology, 2017, 179, 389–398.
15. Milewska A, Zarebski M, Nowak P et al. Human coronavirus NL63 utilizes heparan sulfate proteoglycans for attachment to target cells. J Virol. 2014 Nov;88(22):13221-30. doi: 10.1128/JVI.02078-14. Epub 2014 Sep 3.
16. Wein L, Wein S, Haas SJ et al. Pharmacological Venous Thromboembolism Prophylaxis in Hospitalized Medical Patients. A Meta-analysis of Randomized Controlled Trials. Arch Intern Med. 2007; 167:1476-1486.
17. Pengo V, Cucchini D, Denas G et al. Standardized Low–Molecular-Weight Heparin Bridging Regimen in Outpatients on Oral Anticoagulants Undergoing Invasive Procedure or Surgery. An Inception Cohort Management Study for the Italian Federation of Centers for the Diagnosis of Thrombosis and Management of Antithrombotic Therapies (FCSA). Circulation. 2009; 119:2920-2927.

**APPENDIX I**

**List of participating Centres**

| **Centre**  **#** | **Responsible Investigator** | **Affiliation** | **Contacts** |
| --- | --- | --- | --- |
| **1** | Lucio Brugioni, MD | S.C. Medicina Interna d'Urgenza e Area Critica  Dipartimento di Medicina Interna Generale, d’Urgenza e Post-Acuzie  Azienda Ospedaliero-Universitaria di Modena - Policlinico | [brugioni.lucio@aou.mo.it](mailto:brugioni.lucio@aou.mo.it)  059 4225491 |
| **2** | Enrico Clini, MD, PhD | S.C. Malattie dell’Apparato Respiratorio  Dipartimento di Medicine Specialistiche  Azienda Ospedaliero-Universitaria di Modena - Policlinico | enrico.clini@unimore.it  059 4225922 |
| **3** | Mauro Codeluppi, MD | Unità Operativa di Malattie Infettive  Dipartimento di Onco-Ematologia  Azienda Unità Sanitaria Locale di  Piacenza | m.codeluppi@ausl.pc.it |
| **4** | Cristina Mussini, MD, PhD | S.C. di Malattie Infettive  Dipartimento di Medicine Specialistiche  Azienda Ospedaliero-Universitaria di Modena - Policlinico | cristina.mussini@unimore.it |
| **5** | Giovanni Pinelli, MD | S.C. Medicina Interna d'Urgenza e Area Critica  Dipartimento di Medicina Interna Generale, d’Urgenza e Post-Acuzie  Azienda Ospedaliero-Universitaria di Modena - OCB | pinelli.giovanni@aou.mo.it  059 3961114 |
| **6** | Antonello Pietrangelo, MD, PhD | S.C. Medicina Interna  Dipartimento di Medicina Interna Generale, d’Urgenza e Post-Acuzie Azienda Ospedaliero-Universitaria di Modena - Policlinico  Azienda Ospedaliero-Universitaria di Modena | antonello.pietrangelo@unimore.it  059 4222714 |
| **7** | Andrea Magnacavallo, MD | Unità Operativa Pronto Soccorso, OBI e Medicina d'Urgenza  Dipartimento Emergenza Urgenza  Azienda Unità Sanitaria Locale di  Piacenza | a.magnacavallo@ausl.pc.it  052 3303014 |
| **8** | Matteo Silva, MD | Unità Operativa Medicina D’Urgenza Pronto soccorso OBI  Dipartimento Emergenza Urgenza  Azienda Unità Sanitaria Locale di  Piacenza | m.silva2@ausl.pc.it  052 3303284 |
| **9** | Daniela Aschieri, MD | Unità Operativa Cardiologia Riabilitativa  Dipartimento Emergenza Urgenza  Azienda Unità Sanitaria Locale di  Piacenza | [D.aschieri@ausl.pc.it](mailto:D.aschieri@ausl.pc.it)  052 3880332 |
| **10** | Sergio Orlando, MD | Unità Operativa Medicina Interna Val d’Arda  Dipartimento delle Medicine  Azienda Unità Sanitaria Locale di  Piacenza | [S.Orlando@ausl.pc.it](mailto:S.Orlando@ausl.pc.it)  052 3303314 |

**APPENDIX 2: Timeline-scheme**

| Required investigations or activities | Screening | For any treatment | | | | | | | |  |
| --- | --- | --- | --- | --- | --- | --- | --- | --- | --- | --- |
|  | Within 5 days | Day 1 | Day 2 | Day 3 | Day 4 | Day 5 | Day 7 | Day 8 | Day 9 up to hospital discharge,  or up to occurrence of death | Day 30  Only for patients discharged from hospital before 30 days |
| Clinical history | x | x |  |  |  |  |  |  |  |  |
| Informed consent Form | x |  |  |  |  |  |  |  |  |  |
| **Physical exam** | x | x | x | x | x | x | x | x | x |  |
| **Vital signs*** | x | x | x | x | x | x | x | x | x |  |
| Body weight | x |  |  |  |  |  |  |  |  |  |
| Height | x |  |  |  |  |  |  |  |  |  |
| **Performance status** | x | x |  |  |  |  |  |  | x |  |
| Eligibility criteria | x |  |  |  |  |  |  |  |  |  |
| Pregnancy Test | x |  |  |  |  |  |  |  |  |  |
| Randomization |  | x |  |  |  |  |  |  |  |  |
| **Concomitant medications** | x | x | x | x | x | x | x | x | x |  |
| **Lab Tests**** | x | x | x |  | x |  | x |  | x |  |
| **Enoxaparin (Low-Dose LMWH) every 24 hours** |  | x | x | x | x | x | x | x | x |  |
| **Enoxaparin (High-Dose LMWH) every 12 hours** |  | x | x | x | x | x | x | x | x |  |
| **Health monitoring** |  | x | x | x | x | x | x | x | x | X **^⊥^** |

* systolic and diastolic arterial pressure, heart rate (HR), respiratory rate (RR) and systemic body temperature

** blood cells count, coagulative parameters (INR, PT, aPTT, fibrinogen, D-dimer), parameters for liver (AST, ALT, bilirubin) and renal function (creatinine) and interleukin 6 (IL-6) will be recorded at least at the moment of randomization, after 48 and 96 hours, then according to clinical judgement of the attending physician until to hospital discharge

**^⊥^** Information about these patients will be sought by phone call

**APPENDIX 3: Sepsis Induced Coagulopathy (SIC) score**^1^

| Category | Parameter | 0 point | 1 point | 2 points |
| --- | --- | --- | --- | --- |
| Prothrombin time | PT-INR 4 | <1.2 | >1.2 >1.4 | >1.4 |
| Coagulation | Platelet count (x10^9^/L) | > 150 | < 150 | <100 |
| Total SOFA | SOFA 4 items (respiratory,  cardiovascular, hepatic, renal | 0 | 1 | >2 |

1. Iba T, Nisio MD, Levy JH, et al. New criteria for sepsis-induced coagulopathy (SIC) following the revised sepsis definition: a retrospective analysis of a nationwide survey. BMJ Open 2017;7:e017046. doi:10.1136/bmjopen-2017-017046

**APPENDIX 4: Sequential Organ Failure Assessment (SOFA) score**^1^

| Variables | 0 | 1 | 2 | 3 | 4 |
| --- | --- | --- | --- | --- | --- |
| Central Nervous System  (Glasgow Coma Scale score) | 15 | 13-14 | 10-12 | 6-9 | <6 |
| Respiratory  (PaO_2_ / FiO_2_ ratio) | >400 | ≤ 400 | ≤ 300 | ≤ 200 ^A^ | ≤ 200 ^A^ |
| Cardiovascular, Hypotension  (MAP; mmHg / catecholamine dose; γ/Kg/min) | PAM >70  and NO cathecolamines | PAM < 70  and NO cathecolamines | Dop^B^≤ 5  or  Dob^B^ (any dose) | Dop^B^>5  or  Epi^B^≤0,1  or  Norepi^B^≤0,1 | Dop^B^> 15  or  Epi^B^>0,1  or  Norepi^B^> 0,1 |
| Coagulation  (Platelets ; x 10^3^ u/μL) | >150 | ≤ 150 | ≤ 100 | ≤ 50 | ≤ 20 |
| Liver  (Bilirubin; mg/dl) | <1,1 | 1,2 - 1,9 | 2,0 - 5,9 | 6,0 – 11,9 | >12 |
| Renal  (Creatinine; mg/dl  /diuresis ;ml/24h) | <1,1  +  diuresis > 500 | 1,2 - 1,9  +  diuresis > 500 | 2,0 - 3,4  +  diuresis > 500 | 3,5 – 4,9  or  diuresis <500 | >5  or  diuresis <200 |

A - Values are with respiratory support.

B – Cathecolamines are specified as follow:

| Dop | Dopamine |
| --- | --- |
| Dob | Dobutamine |
| Epi | Epinephrine |
| Norepi | Norepinephrine |

1. Vincent, J. L. *et al.* The SOFA (Sepsis-related Organ Failure Assessment) score to describe organ dysfunction/failure. On behalf of the Working Group on Sepsis-Related Problems of the European Society of Intensive Care Medicine. *Intensive Care Med.***22,** 707–710 (1996).

**APPENDIX 5: flow-chart**

**
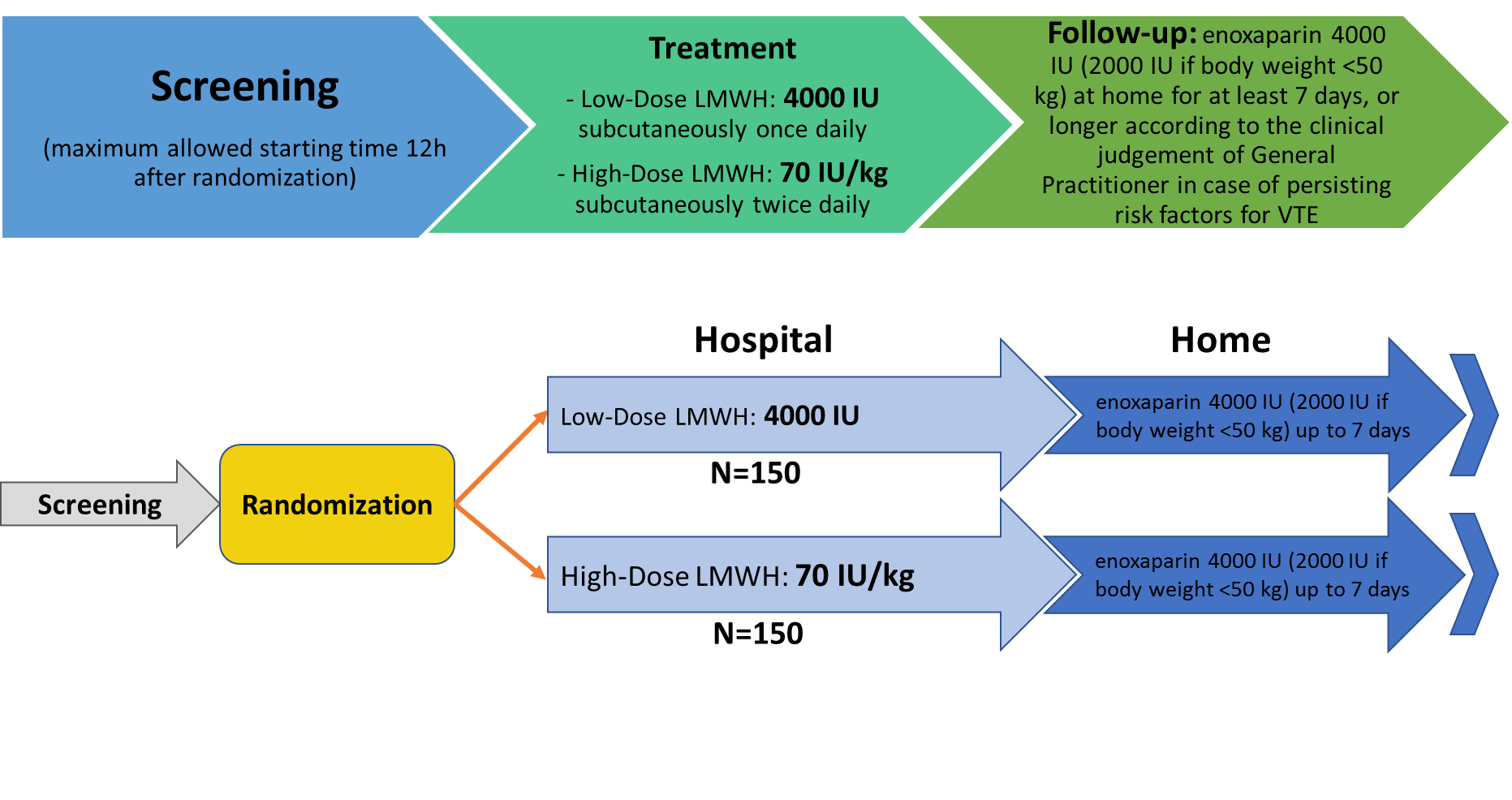
**
